# Enrichment of Olive Fruits in Antioxidant Content by Pre-Harvest Salicylic Acid Treatment

**DOI:** 10.3390/foods9101513

**Published:** 2020-10-21

**Authors:** Gracia Patricia Blanch, María C. Gómez-Jiménez, María Luisa Ruiz del Castillo

**Affiliations:** 1 Instituto de Ciencia y Tecnología de Alimentos y Nutrición, Consejo Superior de Investigaciones Científicas (ICTAN-CSIC), Juan de la Cierva 3, 28006 Madrid, Spain; gblanch@ictan.csic.es; 2 Departamento de Biología Vegetal, Ecología y Ciencias de la Tierra, Universidad de Extremadura, Avda/de Elvas, s/n, 06006 Badajoz, Spain; mcgomez@unex.es

**Keywords:** olives, phytoregulators, polyphenols, free radical scavenging activity, antioxidant, functional foods

## Abstract

We here study the effect of the pre-harvest application of salicylic acid at two different concentrations on the olive phenolic composition. Influence of the cultivar and harvesting day were considered. As a result, the total phenol content increased significantly, particularly when using 200 mg mL^−1^ of salicylic acid. However, the free radical scavenging activity was cultivar dependent. For instance, when the olives were harvested on day 3 and treated with 200 mg mL^−1^ of salicylic acid, the antioxidant activity decreased from 161 to 278 µg mL^−1^ for Arbequina, whereas it increased from 397 to 258 µg mL^−1^ for Picual. Generally speaking, oleuropein and hydroxytyrosol contents enhanced with the application of 200 mg mL^−1^ of salicylic acid. The results found suggest that exogenous salicylic acid is an interesting agronomic practice to enrich olive fruits in antioxidants.

## 1. Introduction

The olive tree (*Olea europaea* L.) has been typical of the Mediterranean region for over 7000 years. Table olives and the oil obtained from them are known worldwide by their nutritional properties. In this regard, both olives and olive oil reduce the risk of heart diseases [1] and lowered cancer risk [2]. Additionally, olive fruits and oil have strong anti-inflammatory properties [3], may protect against ulcers, thanks to their antimicrobial activity, and boost iron intake, which helps regulate immune function and cognitive development [4].

The disease-preventing properties of olive fruits and oil are linked to the occurrence of certain bioactive compounds, mainly the unsaturated fatty acids, oleic and linoleic acids, and some minor components, such as polyphenols. One of the most important polyphenol groups in olive is oleuropein and its related compounds, which are present not only in the fruit but also in all olive tree derivatives [5]. In addition to the nutrition related effects [6], oleuropein and its derivatives also act as natural antioxidants, contributing to the oxidative stabilization of oleic and linoleic acids. However, oleuropein is usually hydrolyzed to tyrosol, which is in turn hydrolyzed to hydroxytyrosol during processing. This leads to a considerable decrease in its content. In particular, the alkaline hydrolysis of oleuropein is essential in table olives to remove the bitterness associated with oleuropein [7] in such a way that eventually the final concentration of oleuropein is frequently rather low [8].

Some researchers have reported the enrichment of olives in polyphenols, particularly in oleuropein, by treatment with polyphenols extracted from olive leaves [9]. This procedure has been applied to enrich table olives, olive oil and other edible vegetable oils in polyphenols. With the same purpose, other procedures have been recently developed as alternatives to the use of olive leave extracts. One of the most used is the application of phytoregulators to the plant. Various compounds have been proved to be efficient as phytoregulators because of their hormonal activity [10]. Nevertheless, scarce studies on the use of phytoregulators in olives have been published to date. In the last few years, our research has been focused on the effectiveness of methyl jasmonate (MJ) and abscisic acid (ABA) as phytoregulators in obtaining enriched olive fruits. In particular, we have studied the effects of the treatment with these two compounds on the content of fatty acids and polyphenols in olives [11,12,13,14]. The results found in these studies reflect that the effect of MJ and ABA on olive phenolics depends on the cultivar and the specific treatment conditions. For this reason, additional phytoregulators that overcome the limitations found for MJ and ABA are still sought.

The purpose of this work was to evaluate a new phytoregulator capable of enriching olive fruits in antioxidant polyphenols overcoming, in turn, the drawbacks of the phytoregulatores tested so far, MJ and ABA. To that end, we assessed the influence of the application of salicylic acid (SA) to the olive trees on polyphenol content. SA has been already used to improve the resistance to root-knot nematodes [15], induce the accumulation of phytoalexins in cotyledons of common bean [16] and promote growth in yarrow [17], among other applications. Regarding its effect on the chemical composition, exogenously applied SA has been reported to increase the content of bioactive compounds in pomegranate [18]. In olive tree, scarce studies on the effect of SA have been carried out. Most of them are mainly focused on the increase in drought adaptability and to the modulation of its physiology and growth responses to drought [19]. All in all, the earlier results on SA published by other researchers [19], together with our own previous experience in phytoregulators, were used as a base to face the present work. Treatments with ABA were simultaneously carried out to be used as a reference.

## 2. Materials and Methods

### 2.1. Chemicals and Samples

MeOH and acetic acid were both provided by VWR Inc. (Bridgeport, PA, USA). Water was obtained from a purification system (Millipore Milford, MA, USA). ABA and SA were purchased by Sigma-Aldrich (Steinheim, Germany) and Acros Organics (Fair Lawn, NJ, USA), respectively. Oleuropein, hyrdroxytyrosol and 1,1-diphenyl-2-picrylhydrazil (DPPH) standards were acquired from Sigma-Aldrich (Milan, Italy), Folin-Ciocalteu reagent from Merck (Darmstadt, Germany) and sodium carbonate from Sigma-Aldrich (Milan, Italy). ABA and SA were applied to Arbequina and Picual olive trees in November and December 2017 at Extremadura University (Badajoz, Spain). Samples were hand-picked randomly. The treatments were accomplished as detailed below.

### 2.2. Treatment of Olive Trees

Two different cultivars (Picual and Arbequina) of olive trees (*Olea europaea* L.) were cultivated in an orchard near Badajoz (Spain). Traditional fertilization: each of N (600 g/tree), P (400 g/tree) and K (500 g/tree) was applied once during initial developmental stage (beginning of March). By using the drip irrigation method, the olive fruits on their branches were carefully irrigated, excluding the rest of the tree. The polyethylene lines with 16 mm in diameter, having in-line drippers at 1.2 m intervals, were installed in the orchard. Each year, the process lasted from May to October. The treatments of fruits were carried out by using 5 trees per treatment of each cultivar. For the experiments, two branches were selected on the basis of uniform size and fruit load. The concentration of ABA used was 100 mg mL^−1^ and two different concentrations of SA (i.e., 100 and 200 mg mL^−1^) were tested on the basis of our previous experience [12,13]. From now on, we will refer to these treatments as ABA, SA100 and SA200, respectively. For each treatment olive fruits contained 5 branches (1 branch per tree) were sprayed and only water was used for controls. The spray of the solutions was carried out at the time of harvest (500 mL per branch). Each of the test trees was separated with at least one guard tree to avoid contamination during the application of the treatment. The absence of wind was considered mandatory to perform the spray. Samples of each cultivar were picked from each tree (i.e., 300 fruits per tree) on two different days of harvesting (days 3 and 6 after treatments). The total number of fruits picked for each treatment and time point of each cultivar was 1500 olives. For the experiments, only samples without any kind of infection of physical injury were included in the study. All fruits were collected by an expert at the maturity stage appropriate for oil processing (i.e., commercial maturity stage). Once the samples were harvested, they were immediately kept in cool bags until analysis, as explained below.

### 2.3. Extraction

The method used to isolate polyphenols is described elsewhere [20]. Each single sample was extracted in duplicate. First of all, the fruits were de-stoned, and the stones were discarded. Subsequently, a 60 mL-volume of 80:20 (*v*/*v*) methanol:water was added to a 5-g weight of the olive pulps. By using an Ultraturrax (IKA, Sigma-Aldrich, Madrid, Spain) the mixture was homogenized and centrifuged (1500 rpm; 10 min) at room temperature. After that, the supernatant was filtered, and the extract was re-extracted. Finally, 30 mL of hexane was added to remove the remaining oil. The hexane layer was discharged and the methanolic extracts were combined, passed through a Whatman No. 1 filter paper and analyzed by High Performance Liquid Chromatography.

### 2.4. Analysis

The extracts obtained were used to determine Total Phenol Content (TPC), the free radical scavenging activity and contents of oleuropein and hydroxytyrosol.

#### 2.4.1. Determination of TPC

A Beckman Coulter DU-800 spectrophotometer (Barcelona, Spain) was used to perform the measurements. The method applied was that previously reported [21]. In brief, a 0.5 mL of Folin–Ciocalteu reagent and 10 mL of sodium carbonate solution (75 g L^−1^) were added to a 0.1-mL volume of the extract. The resulting mixture was made up to 25 mL with distilled water. A blank made by using the same mixture without the reagent was used as a reference. The absorbance was measured at 750 nm against the blank after 1 h. To quantify, a calibration curve made from gallic acid as the standard was utilized. The results were expressed as milligrams of gallic acid equivalents per kg of fruit. Analyses were accomplished in triplicate.

#### 2.4.2. Determination of Oleuropein and Hydroxytyrosol

A Konik-Tech model 560 (Barcelona, Spain) liquid chromatograph (model 7725i, Konik-Tech, Barcelona, Spain) fitted with a manual injector (20-µL sample loop) was used. An a ODS reverse phase (C_18_) column (250 nm × 4.6 mm i.d., 5-µm particle size, ACE, Madrid, Spain) was employed for the separations. The eluent was made up of a mixture of water/acetic acid (95/5, *v*/*v*) and methanol as solvents A and B, respectively. The flow rate was 1 mL/min. The program was as follows: initial composition 95/5% A/B, 85/15 A/B at 3 min, 80/20 A/B at 13 min, 75/25 A/B at 25 min, 70/30 A/B at 35 min, 65/35 A/B at 40 min, 60/40 A/B at 45 min, 55/45 A/B at 47 min, 53/47 A/B at 50 min, 52/48 A/B at 60 min, 50/50 A/B at 64 min, 50/50 A/B at 70 min, 95/5 A/B at 75 min. The chromatographic signals were registered at 280 nm. Blanks between consecutive runs were performed to guarantee the clean-up. Each single sample was run three times. Standard solutions were prepared in 70% (*v*/*v*) methanol to final concentration of 1 mg mL^−1^. Each solution was then diluted to obtain six concentrations of the standard. Calibration curves were established on six data points. Peak areas for the extracts and standards were integrated by use of Konikrom Plus (KNK-725-240) (Konik-Tech, Barcelona, Spain). All analyses were accomplished three times.

#### 2.4.3. 1,1-Diphenyl-2-Picrylhydrazyl FREE Radical (DPPH*) Scavenging Assay

To measure the DPPH activity, a Beckman Coulter DU-800 spectrophotometer (Barcelona, Spain) was used. The method followed was that used by other authors with slight modifications [22]. Each extract was further diluted to final concentrations of 15.6, 62.5, 125, 250 and 500 µg mL^−1^. Then they were put into a 96-well microtiter plate. Prior to DPPH addition, each extraction solution was used as a blank. A 50 µL aliquot of the sample together with 150 µL of DPPH (400 µM) was put into each well. After 30 min of incubation at 37 °C, the diminishment in absorbance at 517 nm was monitored. As a reference, DPPH solution was immediately measured. The percentage inhibition of the DPPH by each dilution was calculated by considering the percentage of the steady DPPH in solution after reaction. Results were expressed as the concentration of extracts that gives rise to a 50% reduction in the DPPH. All the experiments were accomplished in triplicate.

### 2.5. Statistical Analysis

The results are represented as the average of all values together with the standard deviation (±SD). The two days of harvesting and the two olive varieties were included in the statistical study of the data, which were analyzed using one-way analysis of variance (ANOVA). The mean values were compared with the Fisher’s least significant differences (LSD) at the 0.05 probability level.

## 3. Results and Discussion

The treatments of olive trees with ABA and SA were simultaneously carried out [14] on the basis of our previous experience in ABA. Table 1 represents TPC, expressed as mg of Gallic Acid Equivalent (GAE) kg^−1^ in olive fruits from olive trees (*Olea europaea* L.) untreated-control and treated with 100 mg mL^−1^ of ABA, 100 mg mL^−1^ of SA and 200 mg mL^−1^ of SA. For comparison, olive samples were harvested on days 3 and 6 after treatment. Varietal differences were also studied by including two cultivars (Arbequina and Picual). Firstly, TPC values in control olives within the same harvest day were statistically compared and are represented as bold upper-case letters in Table 1. By comparing Arbequina with Picual, no differences in controls were determined on day 3 within the same type of treatment, whereas significant (*p* < 0.05) differences between cultivars were clear within each type of treatment on day 6. In fact, it is interesting to highlight the particularly high TPCs obtained in Picual olives picked on day 6, whose values varied from 1718 to 1901 mg of GAE kg^−1^, as compared with those measured for Arbequina (from 398 to 760 mg of GAE kg^−1^). By comparing the types of treatments within the same cultivar, it is worthy to note the significant (*p* < 0.05) increase observed for controls used in the SA200 treatment in both Arbequina and Picual. Previous studies in our laboratory have already proved higher TPCs in Picual than in Arbequina, although values as high as those here measured for Picual controls on day 6 were never determined [13]. Therefore, the unusually high TPCs for Picual controls on day 6 might be indicative of a possible transference of the phytoregulator used for each treatment from the treated to the control branches. This might also be the reason for the significant increase observed for controls used for SA200 treatment. It is likely that SA200 is so efficient that longer than 3-day harvest times are unnecessary.

The comparison between days of harvesting 3 and 6, represented as normal upper-case letters in Table 1, revealed no significant (*p* ˃ 0.05) differences for control samples, except, as mentioned above, for Picual olives picked on day 6 whose values were particularly high. However, for treated samples, significant (*p* < 0.05) variations between harvest days 3 and 6 were observed for both Arbequina and Picual. It has been described in the literature that TPCs increase as the maturity process progresses up to fully ripe stage, where it starts decreasing [23]. Interestingly, TPCs in SA treated Arbequina olives measured on day 6 did not exceed TPCs on day 3; actually, they dropped. This reflects overripe olive fruits on harvesting day 6 for Arbequina samples, particularly with SA200 treatment. In any case, despite the hypothetical contamination from treated to control branches, Picual control olives harvested on day 6 possess potentially healthier attributes due to their higher TPC. Concerning the effect of the treatments, represented as lower-case letters in Table 1, different results were obtained according to the phytoregulator used. In this regard, by comparing controls and ABA treated samples, the TPC increased significantly (*p* < 0.05) after the exposition of olive trees to ABA in all cases. In contrast to ABA, SA treatments exhibited distinct results depending on the cultivar. For Arbequina, SA was only successful when SA concentration was 200 mg mL^−1^. From Table 1 it can be seen that SA100 treatment only slightly affects TPC in Arbequina olives. However, for Picual, SA100 and SA200 were both efficient, the higher the SA concentration used in the treatment, the higher TPC measured. It is believed that the action mechanism of both ABA and SA is based on the activation of the enzymes involved in the biosynthesis of polyphenols accelerating the maturity process of fruits. In this sense it is possible that the phytoregulator acts as a promotor of phenyl alanine lyase (PAL) activity, which is the first enzyme regulating the phenylpropanoid pathway, which leads to the bioformation of polyphenols. PAL catalyzes the transformation of phenyl alanine into cinnamic acid, which is then converted into naringenin and this latter into different polyphenols [24,25]. In any case, it would be convenient to prove this hypothesis and rule out the activation of any enzyme other than PAL also involved in this pathway.

**Figure 1:**
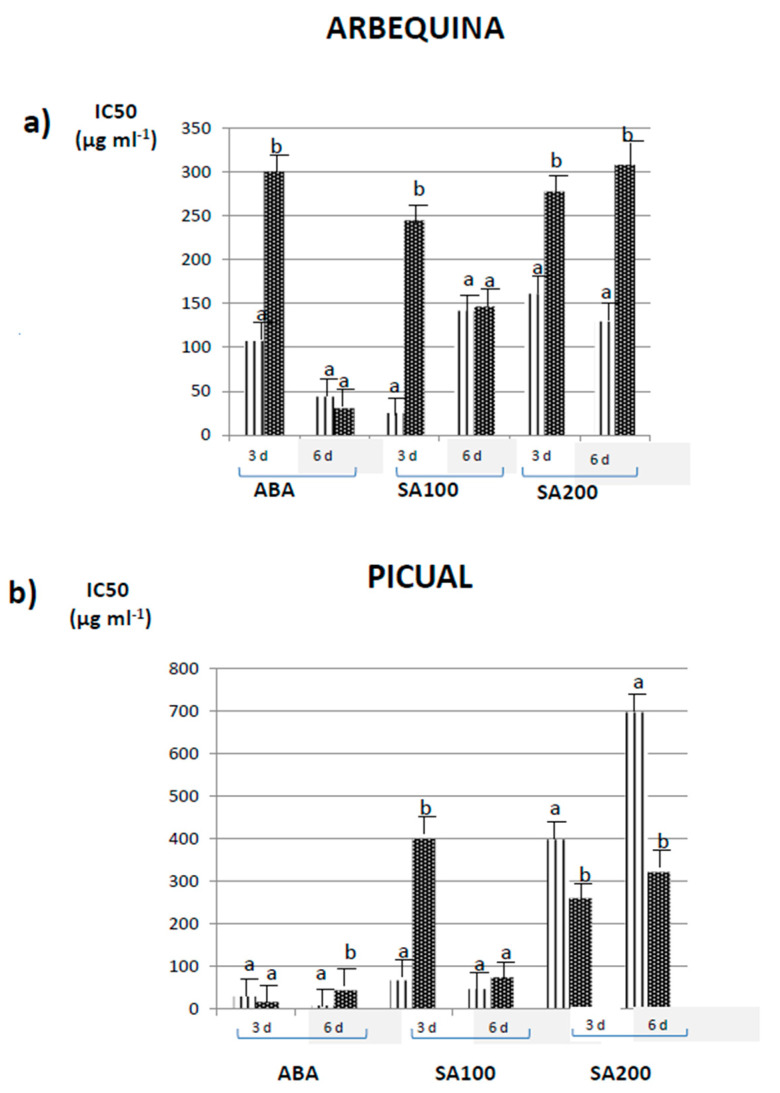
Free radical scavenging radical activity (expressed as IC_50_) of olive fruits control (indicated as striped columns) and treated (indicated as black columns) with ABA, SA100 and SA200 on days 3 and 6 after treatment. Data on Arbequina (**a**) and Picual (**b**) cultivars are included. The values were estimated as means ± Standard Deviation (*n* = 3). Different letters indicate significant (*p* < 0.05) differences between control and treated samples.

Figure 1 indicates the DPPH scavenging activity expressed as IC_50_ (µg mL^−1^) in olive fruits from olive trees (*Olea europaea* L.) untreated-control and treated with 100 mg mL^−1^ of ABA and 100 mg mL^−1^ and 200 mg mL^−1^ of SA. In contrast to TPCs (see Table 1), cultivar differences in the free radical scavenging between Arbequina and Picual controls were apparent in all cases, in particular for controls used in SA200 treatment (i.e., 161 µg mL^−1^ in *Arbequina* vs 397 µg mL^−1^ in Picual on day 3 and 130 vs. 696 µg mL^−1^ on day 6). Regarding the harvest days, DPPH values within the same range were in general terms measured for both 3 and 6 days. A possible correlation between TPC and DPPH results (Table 1 vs. Figure 1) was considered. As a result, the following linear regressions were obtained: y = 0.3114x + 66.74 (*r*^2^ = 0.5052) for Arbequina on day 3, y = 0.0164x + 114.4 (*r*^2^ = 0.0031) for Arbequina on day 6, y = 1.5937x + 674.29 (*r*^2^ = 0.5726) for Picual on day 3, and y = 0.2062x + 690.66 (*r*^2^ = 0.0964) for Picual on day 6. As can be seen, no correlation between TPC and DPPH activity was found. The most interesting aspect to highlight is the fact that the high TPCs estimated for Picual control olives picked on day 6 were not reciprocal to the DPPH activity measured. The lack of correspondence between TPC and DPPH has been observed in olives before [12]. Additionally, some authors have found no relationship between TPC and DPPH values in plant material other than olives [26]. Phenolics constitute one of the most important groups of compounds in plant foods acting as a primary antioxidant. Additionally, they possess ideal structural properties for free radical scavenging activity [27]. The lack of correlation between TPC values and DPPH activity might be due to differences in the polyphenol profile resulting from each treatment. Regarding the effect of the treatments on each cultivar, by comparing controls and treated samples different trends were observed according to the cultivar. For Arbequina, the DPPH activity of controls never improved with any of the treatments. In fact, significant (*p* < 0.05) decreases in the free radical scavenging activity were always measured on day 3 and even on day 6 for SA200 (increase in IC_50_ from 130 in controls vs 308 µg mL^−1^ in treated). In light of these results, ABA followed by SA100 is more recommendable for Arbequina olives since they did not result in a decrease in the DPPH activity as compared with the controls and, in turn, the IC_50_ values obtained were the lowest. However, Picual olives exhibited a different behavior. The antioxidant activity of the controls increased significantly (*p* < 0.05) with SA200 treatment on both picking days 3 and 6. As observed in Figure 1b, IC_50_ values dropped from 397 in the controls to 259 µg mL^−1^ in treated samples, which were measured on day 3, and from 696 in controls to 320 µg mL^−1^ in treated samples on day 6. On the contrary, ABA and SA100 did not provide higher DPPH activity in treated olives as compared with the controls. From these results, SA200 is regarded as more effective for Picual than the other treatments.

All in all, the effect of the treatments was cultivar dependent. For Arbequina, although SA200 was the most effective treatment in terms of TPC; ABA, followed by SA100, was more advantageous because they provided the lowest IC_50_ and, at the same time, satisfactory TPCs, particularly, olives treated with ABA and picked after 6 days. However, for Picual, SA200 was in general more effective in terms of both TPC and DPPH activity. In particular for Picual olives picked on day 3, TPC values ranged from 616 mg of GAE kg^−1^ in the controls to 3354 mg of GAE kg^−1^ in treated samples, and the IC_50_ values varied from 400 µg mL^−1^ in the controls to 259 µg mL^−1^ in treated samples.

Considering the overall results, SA200 treated olives were selected to evaluate the contents of oleuropein. As already mentioned, oleuropein is usually hydrolyzed to other phenolics during processing. For this reason, hydroxytyrosol was included in our study as a reference of the hydrolysis of oleuropein. In any case, both oleuropein and hydroxyrysol are important healthy promoting compounds [28]. From this standpoint, the occurrence of one or another would be equally desirable.

Table 2 and Table 3 depict oleuropein and hydroxytyrosol contents, respectively, expressed as mg and kg^−1^ weight ± SD, in olive fruits from olive trees (*Olea europaea* L.) treated with 200 mg mL^−1^ of SA. Data from olive samples picked on days 3 and 6 after SA200 application and from two cultivars (Arbequina and Picual) are included. Firstly, oleuropein and hydroxytyrosol contents in control olives between cultivars were statistically compared. The results are represented as bold upper-case letters in both Table 2 and Table 3. As observed, oleuropein and hydroxytyrosol contents in the controls did not vary significantly (*p* ˃ 0.05) with the cultivar, except for the oleuropein content in olives picked on day 6, whose content in Picual was statistically higher than in Arbequina (308 vs. 176 mg kg^−1^). These results are in good agreement with those previously described for TPC values (see Table 1). Then, a comparison between the harvest days 3 and 6, represented as normal upper-case letters in Table 2 and Table 3, was carried out. Different behavior with the harvest day was clear according to the cultivar. Specifically, for Arbequina both oleuropein and hydroxytyrosol decreased significantly (*p* < 0.05) from day 3 to 6 in controls, whereas no variation with the harvest day was found for treated samples. In particular, oleuropein decreased in Arbequina controls from 534 mg kg^−1^ on day 3 to 176 mg kg^−1^ on day 6 and hydroxytyrosol from 276 mg kg^−1^ on day 3 to 124 mg kg^−1^ on day 6. The opposite effect was observed for Picual, however, since no significant (*p* < 0.05) variations either in oleuropein or hydroxytyrosol were measured from day 3 to day 6 for controls, while significant (*p* < 0.05) decreases were obtained for treated samples (from 844 mg kg^−1^ on day 3 to 383 mg kg^−1^ on day 6 of oleuropein and from 601 mg kg^−1^ on day 3 to 202 mg kg^−1^ on day 6 of hydroxytyrosol). Concerning the effect of the treatment, expressed as lower-case letters, the exposition of olive trees to SA200 resulted in a significant (*p* < 0.05) increase in oleuropein from 437 to 844 mg kg^−1^ in Picual olives harvested on day 3 and from 176 to 353 mg kg^−1^ in Arbequina olives on day 6. The similar SA200 effect was observed on hydroxytyrosol, whose concentrations increased significantly (*p* < 0.05) from 211 to 601 mg kg^−1^ in Picual olives harvested on day 3 and from 124 to 221 mg kg^−1^ in Arbequina olives harvested on day 6. The results on oleuropein and hydroxytyrosol contents obtained in this study show a variation directly proportional to TPC (Table 2 and Table 3 vs. Table 1). TPCs and oleuropein and hydroxytyrosol contents increased significantly (*p* < 0.05) in Picual fruits on day 3 and in Arbequina fruits on day 6 (and also on day 3) after the application of SA200 to the olive trees. The direct relation between TPC and oleuropein and hydroxytyrosol contents implies once more that there is no relationship between DPPH activity and oleuropein and hydroxytyrosol contents (Table 2 and Table 3 vs. Figure 1), with the exception of SA200 treated Picual olives, for which both the free radical scavenging activity and oleuropein and hydroxytyrosol contents increased with the treatment (see Table 2). In any case, it is important to point out that, in spite of the increase in the DPPH activity of SA200-treated Picual samples with respect to the controls, the absolute IC_50_ values measured were not particularly low.

Oleuropein and hydroxytyrosol are some of the major olive polyphenols and therefore their contribution to TPC is expected. Similarly, their scarce participation in the free radical scavenging activity of the olive extracts also supports reports published in the literature [29]. Particularly, Pellegrini, Visioli, Buratti, and Brighenti, 2001 have found very low oxygen radical absorbance capacity of both pure oleuropein and hydroxytyrosol [29]. In the same line, Zullo and Ciafardini (2008) have described higher antioxidant activity of hydroxytyrosol than that of oleuropein but lower than other polyphenols, such as gallic acid [30]. Additionally, Umeno, Takashima, Murotomi and Nakajima (2015), have reported higher antioxidant efficacy of α-tocopherol and quercetin than those of oleuropein and hydroxytyrosol in olive fruits [28]. In this context, it is necessary to keep in mind that a direct relation between the concentration of a compound and its contribution to the antioxidant activity has not been established. In fact, some minor compounds, even though present in very small quantities, are very active in terms of antioxidant activity. In view of our results, the search for phenolic compounds responsible for the high free radical scavenging activity of the olive extract here found is now scheduled. Nevertheless, even if oleuropein seems to exhibit low DPPH activity, the increase in its content in oil is fundamental because of its known health related properties and its characteristic taste. Besides, oleuropein hydrolysis gives rise to a number of important antioxidant polyphenols such as tyrosol and hydroxytyrosol. On the other hand, the convenience of contrasting several antioxidant methods has been reported since they are based on different mechanisms. For this reason, the determination of antioxidant activity of the oleuropein and hydroxytyrosol extract by 2,2’-azino-bis-3-ethylbenzothiazoline-6-sulfonic acid (ABTS) and fluorescence recovery after photobleaching (FRAP) methods is part of our objective in the near future.

## 4. Conclusions

The exogenous application of olive trees to the phytoregulators, ABA and SA, are useful to obtain functional olives by means of their enrichment in polyphenols, which makes them potentially beneficial to health. Out of the three pre-harvest treatments tested in this research, the application of 200 mg mL^−1^ of SA was the most successful. SA200 treated olives exhibited higher TPC levels, oleuropein and hydroxytyrosol contents and higher antioxidant capacity in terms of free radical scavenging activity for Picual olives. The objective is now to expand this study to the search for additional phenolics and non-phenolics which contribute to the antioxidant activity here measured. In addition, ABTS and FRAP methods will be carried out along with DPPH. Finally, the potential residuals of SA will be analytically determined in the treated olive fruits to guarantee the safety of the proposed method and, therefore, its transference to the industry.

## Figures and Tables

**Table 1:** Total phenol content (mg of Gallic Acid Equivalent (GAE) kg^−1^) of olive fruits from olive trees (*Olea europaea L.*) control and treated with abscisic acid (ABA) and salicylic acid (SA).

Cultivar	Arbequina			Picual		
Treatment	ABA	SA100	SA200	ABA	SA100	SA200
Harvest Day 3						
Control	418 ± 0.6 ^AAa^	460 ± 0.5 ^AAa^	708 ± 0.8 ^BAa^	536 ± 0.6 ^AAa^	424 ± 0.5 ^AAa^	616 ± 0.7 ^BAa^
Treated	992 ± 0.8 ^Ab^	654 ± 0.7 ^Aa^	4629 ± 1.2 ^Ab^	1180 ± 0.8 ^Ab^	808 ± 0.9 ^Ab^	3718 ± 1.1 ^Ab^
Harvest Day 6						
Control	562 ± 0.7 ^AAa^	398 ± 0.6 ^AAa^	760 ± 0.8 ^BAa^	1718 ± 0.9 ^CBc^	1809 ± 0.7 ^CBa^	1901 ± 0.8 ^CBa^
Treated	1322 ± 0.9 ^Bb^	435 ± 0.7 ^Ba^	1763 ± 0.9 ^Bc^	2445 ± 1.0 ^Bd^	3019 ± 1.2 ^Bb^	3354 ± 1.0 ^Ab^

Data are presented as means resulting from three independent samples. Different superscript bold upper-case letters in the same row within the control samples and the same harvest day indicate differences at *p* < 0.05. Different superscript normal upper-case letters in the same column between harvest days 3 and 6 within the same type of sample (control and treated), same treatment and same cultivar indicate differences at *p* < 0.05. Different superscript lower-case letters in the same column between control and treated samples within the same harvest day, same treatment and same cultivar indicate differences at *p* < 0.05.

**Table 2:** Oleuropein contents (expressed as mg kg^−1^ weight ± SD) in olive fruits from olive trees (*Olea europaea* L.) treated with 200 mg mL^−1^ of salicylic acid (i.e., SA200).

SA200 Treatment		
Cultivar	Arbequina	Picual
Harvest Day 3		
Control	534 ± 0.8 ^AAa^	437 ± 0.9 ^AAa^
SA200-Treated	310 ± 0.6 ^Ab^	844 ± 0.8 ^Ab^
Harvest Day 6		
*Control*	176 ± 0.5 ^ABa^	308 ± 0.8 ^BAa^
*SA200-Treated*	353 ± 0.9 ^Ab^	383 ± 0.7 ^Ba^

Data are presented as means of three independent samples. Different superscript bold upper-case letters in the same row between cultivars within the control samples and the same harvest day indicate differences at *p* < 0.05. Different superscript normal upper-case letters in the same column between harvest days 3 and 6 within the same type of sample (control and treated) and same cultivar indicate differences at *p* < 0.05. Different superscript lower-case letters in the same column between control and treated samples within the same harvest day and same cultivar indicate differences at *p* < 0.05.

**Table 3:** Hydroxytyrosol contents (expressed as mg kg^−1^ weight ± SD) in olive fruits from olive trees (*Olea europaea* L.) treated with 200 mg mL^−1^ of salicylic acid (i.e., SA200). Data from olive samples picked on days 3 and 6 after SA200 application and from two varieties (Arbequina and Picual) are included.

SA200 Treatment		
Cultivar	Arbequina	Picual
Harvest Day 3		
Control	276 ± 0.7 ^AAa^	211 ± 0.7 ^AAa^
SA200-Treated	271 ± 0.9 ^Aa^	601 ± 0.8 ^Ab^
Harvest Day 6		
Control	124 ± 0.7 ^ABa^	148 ± 0.5 ^AAa^
SA200-Treated	221 ± 0.8 ^Ab^	202 ± 0.6 ^Ba^

Data are presented as means of three independent samples. Different superscript bold upper-case letters in the same row between cultivars within the control samples and the same harvest day indicate differences at *p* < 0.05. Different superscript normal upper-case letters in the same column between harvest days 3 and 6 within the same type of sample (control and treated) and same cultivar indicate differences at *p* < 0.05. Different superscript lower-case letters in the same column between control and treated samples within the same harvest day and same cultivar indicate differences at *p* < 0.05.
